# Satellite-based estimates of groundwater storage depletion over Egypt

**DOI:** 10.1007/s10661-023-11171-3

**Published:** 2023-04-20

**Authors:** Ahmed Shalby, Sobhy R. Emara, Mohammed I. Metwally, Asaad M. Armanuos, Doaa E. El-Agha, Abdelazim M. Negm, Tamer A. Gado

**Affiliations:** 1 Faculty of Engineering, Tanta University, Tanta, 31733 Egypt; 2 Faculty of Engineering, Suez University, Suez, 43512 Egypt; 3 Faculty of Engineering, Zagazig University, Zagazig, 44519 Egypt

**Keywords:** GRACE, Groundwater monitoring, Aquifer storage, GLDAS, Remote sensing

## Abstract

An arid climate accompanied by a freshwater shortage plagued Egypt. It has resorted to groundwater reserves to meet the increasing water demands. Fossil aquifers were lately adopted as the sole water source to provide the irrigation water requirements of the ongoing reclamation activities in barren areas. Yet, the scarcity of measurements regarding the changes in the aquifers’ storage poses a great challenge to such sustainable resource management. In this context, the Gravity Recovery and Climate Experiment (GRACE) mission enables a novel consistent approach to deriving aquifers’ storage changes. In this study, the GRACE monthly solutions during the period 2003–2021 were utilized to estimate alterations in terrestrial water storage (TWS) throughout Egypt. Changes in groundwater storage (GWS) were inferred by subtracting soil water content, derived from the GLDAS-NOAH hydrological model, from the retrieved TWS. The secular trends in TWS and GWS were obtained using the linear least square method, while the non-parametric technique (Mann–Kendall’s tau) was applied to check the trend significance. The derived changes in GWS showed that all aquifers are undergoing a significant loss rate in their storage. The average depletion rate over the Sinai Peninsula was estimated at 0.64 ± 0.03 cm/year, while the depletion rate over the Nile delta aquifer was 0.32 ± 0.03 cm/year. During the investigated period (2003–2021), the extracted groundwater quantity from the Nubian aquifer in the Western Desert is estimated at nearly 7.25 km^3^. The storage loss from the Moghra aquifer has significantly increased from 32 Mm^3^/year (2003–2009) to 262 Mm^3^/year (2015–2021). This reflects the aquifer exposure for extensive water pumping to irrigate newly cultivated lands. The derived findings on the aquifers’ storage losses provide a vital source of information for the decision-makers to be employed for short- and long-term groundwater management.

## Introduction

Globally, groundwater has become an essential freshwater source for agriculture and domestic supply in many countries. Consequently, groundwater stores are experiencing increasing demands getting a global withdrawal rate of 750–800 km^3^/year (Frappart & Ramillien, 2018). In some regions, especially where groundwater-based irrigation is intensive, groundwater pumping rates exceed renewability rates, and hence both aquifer’s storage and water tables dropped dramatically. Accordingly, groundwater depletion has been reported in many regions worldwide and negative consequences of over-pumping become inevitable (Konikow & Kendy, 2005; Wada et al., 2012). Richey et al. (2015) stated that 21 aquifers, out of the 37 world’s major aquifers, are being stressed. The most stressed aquifers that pose an urgent depletion risk include the California central valley aquifer (USA), Murzuk-Djado Basin (Maghreb region), Arabian aquifer Basin (Arabia Gulf region), Indus Basin (India), and North China aquifer (China). In cases of non-renewable aquifers, where recharge is limited or null, permanent groundwater mining constitutes depletion. While in replenished aquifers, depletion is indicated by substantial head declines (Bartolino & Cunningham, 2003). This would result in a major environmental crisis unless countermeasures are implemented in time. Accordingly, it is important to quantify rates of groundwater depletion to mitigate the associated hazards. Yet, monitoring aquifers storage remains a challenge in many regions owing to a lack of shared data about monitoring networks and the absence of water pumping reports. Globally, measurements regarding the variability of groundwater storage (GWS) are extremely scarce (Konikow & Kendy, 2005). The in-situ measurements, if exist, are often characterized by data inconsistency, and spatial and temporal gaps, and often reflect only local estimates of GWS.

Reliable and periodic observations of aquifer heads (piezometric data) are required to estimate the changes in GWS over time. Fortunately, using the Gravity Recovery and Climate Experiment (GRACE) satellite-acquired information provide inferring the alterations in the soil water masses. GRACE mission is the first remote sensing platform to enable consistent monitoring of terrestrial water storage (TWS) (Li et al., 2012). GRACE satellites were launched in 2002 and continued working until 2017. To follow GRACE’s successful predecessor, a novel mission, GRACE follow-on, has been launched in 2018 (Richey et al., 2015). The GRACE satellites detect each tiny acceleration in the Earth’s gravity field which is mainly attributed to the redistribution of TWS (Frappart & Ramillien, 2018). Groundwater component naturally accounts for the majority of TWS. Accordingly, the GRACE-based anomaly of TWS can be employed to estimate variations in GWS by isolating other terrestrial water mass components (e.g., surface water, soil moisture, and snow). Indeed, groundwater engineering is of the last hydrological sciences to benefit from remote sensing. The GRACE dataset has become the only hope to measure the temporal variations of GWS in data-poor regions all over the world. During the last decades, GRACE has been proven reliable as an efficient method for monitoring storage dynamics and availability (Rodell et al., 2007). GRACE is promising as it is available for virtually all aquifers with unprecedented accuracy and sufficient resolutions. Thanks to the constantly improving and advancement of GRACE data processing approaches, GRACE spatial resolution has become a few hundred kilometers. Applying the recent GRACE release (RL 06), the changes of 1.5 cm in the TWS, measured as Equivalent Water Height (EWH), over a watershed area of 200,000 km^2^ could be detected (Sun et al., 2010). The error of the GRACE land water solutions is a few millimeters of EWH, ranging from 15 mm in the polar regions to around 40 mm at the Earth’s Equator. (Swenson et al., 2006).

The unique potential of the GRACE technique to monitor changes in GWS has been confirmed in many studies (e.g., Moiwo et al., 2009; Strassberg et al., 2007; Swenson et al., 2008; Yeh et al., 2006). In these studies, a close matching in pattern was demonstrated when comparing the GRACE-derived GWS variations with those based on in situ water level observations. For instance, Yeh et al. (2006) concluded a reasonable coincidence between the seasonal amplitude of GRACE-estimated GWS and those of the in-situ observations in Illinois, the USA. Strassberg et al. (2007) demonstrated a robust correlation between seasonal measured and GRACE-derived GWS variations in the high plains aquifer, USA. Swenson et al. (2008) observed a consistent phase between the annual variation of well-level data and GRACE estimation of groundwater anomalies. Moiwo et al. (2009) found a consistent agreement between the hydrological measurements and GRACE-estimated data in the Hai River basin, China. The comparison affirmed that GRACE has sufficiently characterized storage change in such a semi-arid watershed indicating an annual loss rate in the range of 12.72 to 23.76 mm.

Recently, the GRACE dataset, along with external information to disaggregate the four terrestrial water components, has been widely utilized for monitoring GWS changes in major world aquifers (e.g., Ahmed et al., 2016; Fallatah et al., 2017; Othman et al., 2022). It has been used to define trends and infer rates of groundwater depletion and aquifer stress indices, with promising results. Jiao et al. (2015) analyzed the release 05 of GRACE-based TWS anomalies from 2003 to 2012 in the Badain Jaran desert (China) which was represented as nine grids. Changes in GWS were isolated by subtracting TWS from soil moisture anomalies simulated by GLDAS, while the contribution of surface runoff and snow water was negligible. The results demonstrated a gradual decrease in GWS with an average depletion rate of 6.54 mm/year, which led to a significant shrinkage of the spring area. Changes in GWS throughout the San Joaquin River Basins, USA from 2003 through 2010 showed a loss rate of 31.0 ± 2.7 mm/year (Famiglietti et al., 2011). Satellite-based and in situ observations of surface water, snow, and soil moisture were combined to isolate the groundwater contribution to the GRACE-TWS anomalies. Mohamed (2020) integrated outputs of the community land surface model with GRACE estimations to measure the storage variations over the Nile Delta aquifer, a large water-bearing storage in Egypt. The aquifer is subjected to groundwater depletion varying from (−2.11 ± 0.85) mm/year in (2003–2006) to (−5.8 ± 1.74) mm/year in (2009–2012). The author tried to verify the results against limited field data showing an acceptable skill of the GRACE technique to capture the long-term changes in the aquifer’s storage.

Egypt is increasingly dependent on groundwater to compensate for the freshwater availability gap. Thus, groundwater is pumped, with no rationing restrictions, to fulfill agricultural water demands in different areas. Fossil desert aquifers are now being exploited to supply the bulk of the water required to irrigate newly cultivated areas in the barren lands within the recently advocated rural development project (SIS, 2016). The growing exploitation of groundwater resources for irrigation is unavoidable to provide sufficient food production for the growing population. Yet, intensive pumping in these areas leads to severe groundwater drawdown in renewable aquifers and increases the susceptibility of non-renewable aquifers to depletion. Shifting the current pumping policy to achieve sustainable water resources management requires the integration of multiple sourced-information about aquifers storage. In Egypt, there are insufficient ground-based networks to monitor GWS variations and even the pumping reports are considered sensitive or confidential. This paper addresses a framework for tracking the depletion of Egyptian groundwater reserves which are subject to unrestricted water withdrawal. Specifically, the study will estimate aquifer storage loss rates in the last 2 decades by applying the GRACE‐based approach. Addressing the time series of the aquifer storage is essential to mitigate the adverse consequences of over-pumping and to provide reliable information to improve groundwater exploitation policies.

## Main groundwater reserves and abstraction fields in Egypt

In Egypt, there are six aquifers of high potentiality for exploitation, as shown in Fig. 1. Although this study covers the spatial extension of Egypt, it is more concerned with the three major aquifers (i.e., the Nile Delta aquifer, the Moghra aquifer, and the Nubian sandstone aquifer) where groundwater is exploited on a large scale. The characteristics of deposits formation of these aquifers are described in the following:

1. The Nile Delta aquifer, a huge renewable groundwater reserve, covers the Nile Delta region and the adjacent desert fringes, with an area of about 32,650 km^2^ (Armanuos & Negm, 2019), representing about 4% of the country’s total area. The aquifer forms originally from confined Pleistocene gravel and sand deposits overlayed by clay sediments from the Holocene (Sefelnasr & Sherif, 2014). The depth of the aquifer increases from 200 m in the south to 1 km in the northern parts (Armanuos & Negm, 2019). The depth to water level increases from 2 m in the northern portions to about 4 m in the middle portions and reaches up to 5 m in the southern (Morsy, 2009; RIGW, 2002). The aquifer capacity is estimated to be about 500 × 10^9^ m^3^, and it is mainly exploited for agriculture and other domestic purposes (Ahmed et al., 2022). The aquifer salinity dropped from 5000 ppm in the northern margins to 1500 ppm in the southern portions (Abd-Elhamid et al., 2019). The aquifer water budget balance decreased from + 3.98 km^3^/year in 1981 (Kashef, 1983), to + 2813.984 Mm^3^/year in 1990 (Dahab, 1993). In the last decades, abstraction rates have increased dramatically (Armanuos & Negm, 2019). Accordingly, the aquifer budget balance turned into a negative balance (Negm et al., 2019), which is affirmed by the observed drawdown in the water head throughout the aquifer.
2. The Moghra aquifer in the north of the Western Desert, a mixture of renewable and fossil water, covers the Moghra desert area at the western edge of the Nile Delta with an area of 50,000 km^2^ (El Tahlawi et al., 2008). The aquifer consists mainly of Miocene fluviatile and fluvio-marine gravel and coarse sand sediments with clay and siltstone intercalations (Sayed et al., 2020). The aquifer depth reaches up to 900 m in the center and decreases towards the north and west, where its base is in line with the ground level (Gomaa et al., 2021). The aquifer is marginally replenished, with low water quantities, specifically from (i) the Nile Delta aquifer through seepage, (ii) the overlying aquifers (Miocene limestone aquifer and Nubian Sandstone Aquifer System) through upward leakage, and (iii) a minor contribution from rainfall through infiltration. The main aquifer discharge source is evaporation from local depressions (i.e., Qattara in the west and Wadi El-Natroun in the east) and by lateral seepage into carbonate formations (Abdel Mogith et al., 2013; Morad et al., 2014). The aquifer water salinity ranges, from slightly brackish (1000 ppm) within a narrow wedge in the vicinity of the Nile delta to saline (up to 12,000 ppm) over the rest of the aquifer’s area (Eltarabily & Moghazy, 2021). The Moghra desert is one of the areas selected for the national land reclamation project that was inaugurated in late 2015. Around 200 Mm^3^ of water will be annually abstracted to sustain ongoing cultivation and fish breeding activities (Sayed et al., 2019).
3. The Nubian sandstone aquifer cover most (83%) of the country’s whole area (Sharaky et al., 2019). The aquifer occurs in central and northern Egypt where it is confined by a thick Upper Cretaceous shale deposit separating it from fissured carbonate aquifers (Fig. 1). It overlays the Proterozoic basement rocks, and its total thickness ranges from 0.5 to 4 km (Aly et al., 2019). The aquifer system is composed mainly of Paleozoic and Mesozoic sandstone, intercalated by pre-Upper Cretaceous clay and shale deposits (Aly et al., 2019). It consists of two major formations (i.e., the post-Nubian and the Nubian reservoirs) separated by a low-permeability layer (i.e., aquitard). Its groundwater quality is relatively good: fresh (total dissolved solids (TDS) less than 1000 mg/L) in the Western Desert, slightly brackish (1500 < TDS < 2000 mg/L) in the Sinai Peninsula and brackish (3000 < TDS < 4000 mg/L) in the Eastern Desert (Elmansy et al., 2020). It is a high-potential aquifer in Egypt, water stored in the aquifer is about 40,000 km^3^ of paleowater (CEDARE, 2014).
  In the southern portions of the Western Desert, sandstone outcrops expose, and the aquifer is unconfined. This has fostered extensive development to have occurred in the southwestern part of the country, where this study scope is. Originally, the aquifer in this region supplied the New Valley Oases (Kharga, Dakhla, Bahareya, and Farafra) through free-flowing springs and tube wells. These shallow wells have run dry since 1960 and have been replaced by deep wells that were installed for the extensive irrigation project that was developed in the vicinity of old oases as well as “East Oweinat,” and “Darb El-Arbain” areas. The cultivation area steadily increased to reach 4200 ha in 2003 with an estimated total annual extraction of 2.8 Km^3^ (CEDARE, 2014). By 2015, the extraction rate has increased by 500% (Ebraheem et al., 2002). Consequently, groundwater levels have fallen over the last 40 years by 60 m within the wells fields (El-Rawy & Smedt, 2020). According to the recently released national groundwater management policies, large cultivation schemes are being developed in this region where the role of the Nubian aquifer has been given more emphasis.
  The Cretaceous Nubian Sandstone is the main aquifer within the desert of the Sinai Peninsula, Fig. 1. Its depth varies from 100 m in the central portion to 500 m in the south (Ahmed, 2020). Surface recharge, from rainfall, occurs over zones of sandstone outcrops at the mountain’s foothills and fractured depositions (Balderer et al., 2014). The Mediterranean Sea in the north and the Red Sea’s gulfs in the south (i.e., the Suez gulf to the west and the Aqaba Gulf to the east) represent natural effluents of the groundwater reserves of the Sinai Peninsula (Ahmed et al., 2022).

**Fig. 1:**
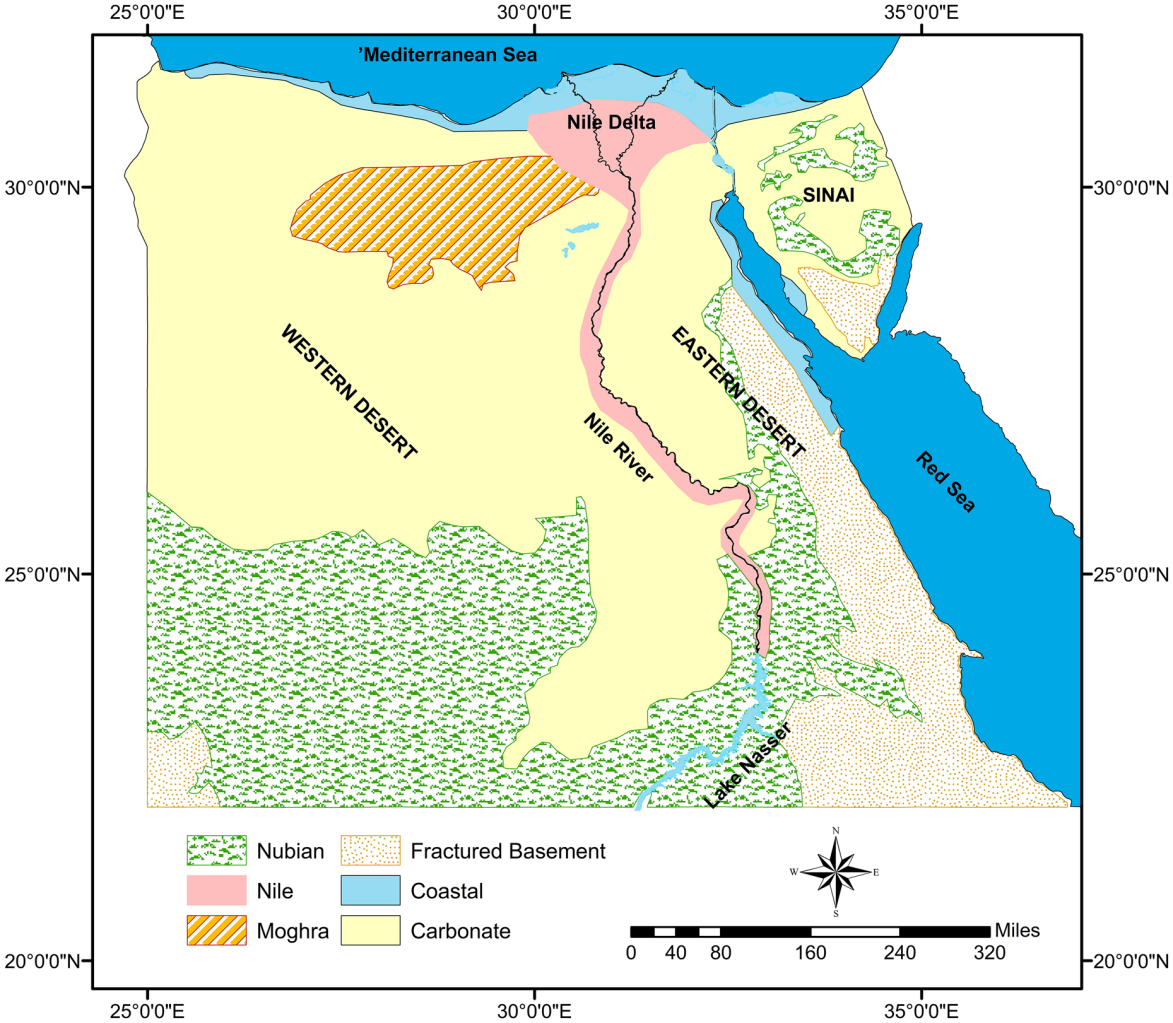
Spatial distribution of the physiographical regions and the major aquifer systems in Egypt

## Methodology

The applied approach in this research is upon using GRACE processed data to track trends in Egypt’s groundwater storage. The temporal monthly TWS changes, mass deviation from the baseline, are expressed in terms of EWH and posted on a 1° grid resolution. The other approach of deriving regional mass concentration (Mascon) from orbit perturbation data is complicated to be realized, albeit its higher precision (Mohamed et al., 2023). In the following sections, more details about data processing and extraction as well as procedures for deriving the direction and degree of changes in groundwater storage are discussed.

### Approach of using GRACE data

Equation (1) is the starting point for recovering surface mass changes from the GRACE monthly solutions (Cheng et al., 2011). Yet, the spherical harmonic errors in the results become larger for higher degrees. Gaussian spatial filter with a radius of 300 km is to be applied to diminish the spherical harmonic coefficients of high degrees (Jekeli, 1981)

1 $$\begin{aligned}\begin{aligned}\Delta h\left(\theta,\;\varphi\right)=& \frac{\alpha\;\rho_{ave}\pi}{3\;\rho_{wat}}\sum_{l\;=\;0}^\infty\;\sum_{m\;=\;0}^l\;{\widetilde P^m}_l\;\left(\mathrm{Cos}\;\theta\right)\frac{2l+1}{1+k_i}\\&\times\left(\Delta C_l^m\;\left(m\phi\right)+\Delta S_l^m\;\mathrm{Sin}\left(m\phi\right)\right)\end{aligned}\end{aligned}$$

where *Δh (θ, φ)* is the EWH changes, α is the mean radius of the planet, P_l_^m^ are normalized associated legendre functions, θ and φ are the co-latitude and longitude, k_l_ is the l^th^ degree Love number, *ΔC*_*l*_^*m*^ and *ΔS*_*l*_^*m*^ are temporal changes in the spherical harmonic coefficients in which m and l are the order and degree respectively, ρ_ave_ is the mean density of the planet and ρ_wat_ is the freshwater density.

Equation (2) shows the Gaussian smoothing function (*W*_*l*_ ) to smooth the gravity field. And the Gaussian kernel function W(α) is stated in Eq. (3).

2 $$\begin{aligned}W_l=\int\limits_0^\pi W(\alpha)\times P_l(\alpha)\times\mathrm{Sin}\left(\alpha\right)\;d\;\alpha\end{aligned}$$

3 $$\begin{aligned}W\left(\alpha\right)=\frac{In\;\left(2\right)}{2\pi\left[1-\mathrm{Cos}\left(r/\alpha\right)\right]}\frac{\exp\left[-b\left(1-\mathrm{Cos}\;\alpha\right)\right]}{1-e^{-2b}}\end{aligned}$$

Additionally, postprocessing is then applied to obtain the GRACE solutions (Landerer & Cooley, 2021): (1) The influences of the tides (i.e., ocean, and solid earth pole) and nontidal (i.e., atmospheric) signals are removed by applying a background model (Landerer & Swenson, 2012); (2) The effect of the correlated errors was minimized using a decorrelated (de-striping) filter (Swenson & Wahr, 2006); (3) Ellipsoidal correction suggested by Ghobadi-Far et al., (2019) has been applied; (4) Glacial isostatic adjustment (GIA) using the GIA model denoted as “ICE-6G_D” by (Richard Peltier et al., 2018); (5) Optimizing correction approach of annual variations and trends in the geocenter motion (Sun et al., 2016); (6) Finally, due to the uncertainty in C20 (degree 2 order 0) estimated by GRACE, It was replaced with the values obtained from satellite laser ranging (Loomis et al., 2019).

### Data retrieving

TWS anomalies are processed and made available by three official processing centers (GRACE science data centers): “the Center for Space Research (CSR) in Austin, Texas, United States, the Jet Propulsion Laboratory (JPL) in Pasadena, California, United States, and the GeoForschungs Zentrum (GFZ) in Potsdam, Germany” (Gemitzi & Lakshmi, 2018). There is a slight difference in the gravity field solution strategies and the processing technique applied by each centre (Mohamed, 2020). Free access to online data is provided by the Physical Oceanography Distributive Active Archive Center (PODAAC), where the data are available in netCDF, ASCII, and GeoTIFF formats. In this study, GRACE data release-06 version 04 (RL06V04) spanning the period from January 2003 to December 2021 was retrieved. This data set constitutes a considerable improvement over other releases thanks to advancements in the applied processing techniques (Landerer & Swenson, 2012), as described in “Approach of using GRACE data” section of this article. Eighty-nine monthly GRACE solutions were extracted from the three processing centers, and their scaled solutions were averaged and posted in a geographic information system (GIS) platform. Grids of EWT variation were then generated and projected onto the WGS 1984, UTM Zone 36N coordinate system on the GIS environment. Afterwards, the area-averaged storage anomaly was inferred from the generated raster maps applying re-truncation to each aquifer extent. This approach (area-weighted averaging) is necessary to support a sufficient and consistent spatial extent display of the aquifers’ storage changes.

### Estimating groundwater storage

A straightforward technique for estimating the variations in GWS (${\Delta }\,{\text{W}}_{{{\text{Groundwater}}}}$) is to isolate the included contributions from the other hydrological components, i.e., surface water (${\Delta }\,{\text{W}}_{{\text{Surface Water}}}$), soil water (${\Delta }\,{\text{W}}_{{{\text{Soil}}}} \,_{{{\text{Water}}}}$), and snow (${\Delta }\,{\text{W}}_{{{\text{Snow}}}}$), from GRACE-based anomalies of TWS (${\Delta }\,{\text{TWS}}$), Eq. (4). In areas that are featured by arid climates and the absence of snow, the contribution of snow is negligible. Hence, the terrestrial-based water balance formula is equated with the changes in surface water, soil moisture, and groundwater. Furthermore, due to the marginal annual alteration in canal water levels throughout the case study, the effect of change in the surface water is neglected. The fixed water levels in the irrigation canal network throughout the Nile Valley and Delta are regulated by the High Aswan dam and successive hydraulic structures including regulators and barrages. Thus, changes in GWS are inferred by removing the ${\Delta }\,{\text{W}}_{{{\text{Soil}}}} \,_{{{\text{Water}}}}$ content from the GRACE-derived TWS anomaly, Eq. (5). Since the lack of available monitoring networks, the GLDAS-NOAH land surface model is used instead to provide information on water in the soil. The GLDAS hydrological model provides a large quantity of data including total column soil moisture at several depths up to 200 cm (Rodell et al., 2007). The soil moisture outputs from the “GLDAS_NOAH10_M_2.1” model have a spatial resolution equal to a degree, and its temporal resolution is monthly. The soil moisture (SM) is extracted for four layers (0–10 cm, 10–50 cm, 50–100 cm, and 100–200 cm) to represent the unsaturated water mass change. Storage of the SM in the top two meters of the soil is stable, and the amplitude within the four layers is the same, but a fairly dampening is recognized at the upper 50 cm where shallow-rooted crops are penetrating. To be consistent with the anomaly-derived monthly series of the TWS, the SM data were converted into anomaly data. Accordingly, the relative time-average values from 2004 to 2009 were subtracted from the original data outputted from the GLDAS NOAH model.

4 $$\begin{aligned}\Delta W_{Groundwater}=&\ \Delta TWS-\Delta W_{Surface\;Water}\\&-\Delta W_{Soil\;Water}-\Delta W_{Snow}\end{aligned}$$

5 $$\begin{aligned}\Delta W_{Groundwater}=\mathrm\Delta TWS-\mathrm\Delta W_{Soil\;Water}\end{aligned}$$

### Trend analysis

Secular trends of the time series were obtained by the linear least square fitting method (linear regression analysis). Equation (6) was used to calculate the slope value of the best-fit line for a series of points [(*x*_1_, *y*_1_), (*x*_2_, *y*_2_), …., (*x*_*n*_, *y*_*n*_)], where *x* is the independent variable with an average of $\overline{x}$ and *y* is the dependent random variable with an average of $\overline{y}$.

6 $$\begin{aligned}Slope=\frac{{\mathop{\sum}\limits_{i\;=\;1}^n}(y_i-\overline{y})(x_i-\overline{x})}{{\mathop{\sum}\limits_{i\;=\;1}^n}{(x_i-\overline{x})}^2}\end{aligned}$$

The residual error at each point is calculated as $$e_{i} = y_{i} - \overset{\lower0.5em\hbox{$\smash{\scriptscriptstyle\frown}$}}{y}_{i}$$ representing the distance that the observed value $$(y_{i} )$$ deviated from the estimated value by the regression model $$(\overset{\lower0.5em\hbox{$\smash{\scriptscriptstyle\frown}$}}{y}_{i} )$$. The standard residuals of the regression slope are estimated as in Eq. (7) representing the average distance at all points along the fit line.

7 $$\begin{aligned}&Standard\;Error\;of\;Regressions\;Slope\\&\quad=\sqrt{\frac{{\mathop{\sum}\limits_{i\;=\;1}^n}\left(y_i-{\overset\frown y}_i\right)}{n-2}}\Bigg/\sqrt{{(x_i-\overline x)}^2}\end{aligned}$$

The generated trends were then statistically investigated using the Mann–Kendall’s tau method (Kendall, 1975; Mann, 1945) to identify which ones are statistically significant at 95% (*α* = 5%) and 90% (*α* = 10%) confidence levels. The approach of Mann–Kendall’s tau, a non-parametric technique, is widely used for trend analysis because it does not assume any specific theoretical distribution of the random variables. In this approach, the null hypothesis (H_0_) states that there is no significant trend in the investigated time series, which is rejected when the *p*-value is less than α, indicating a statistically significant trend (positive or negative).

## Results and discussion

### Temporal variations in TWS

**Fig. 2:**
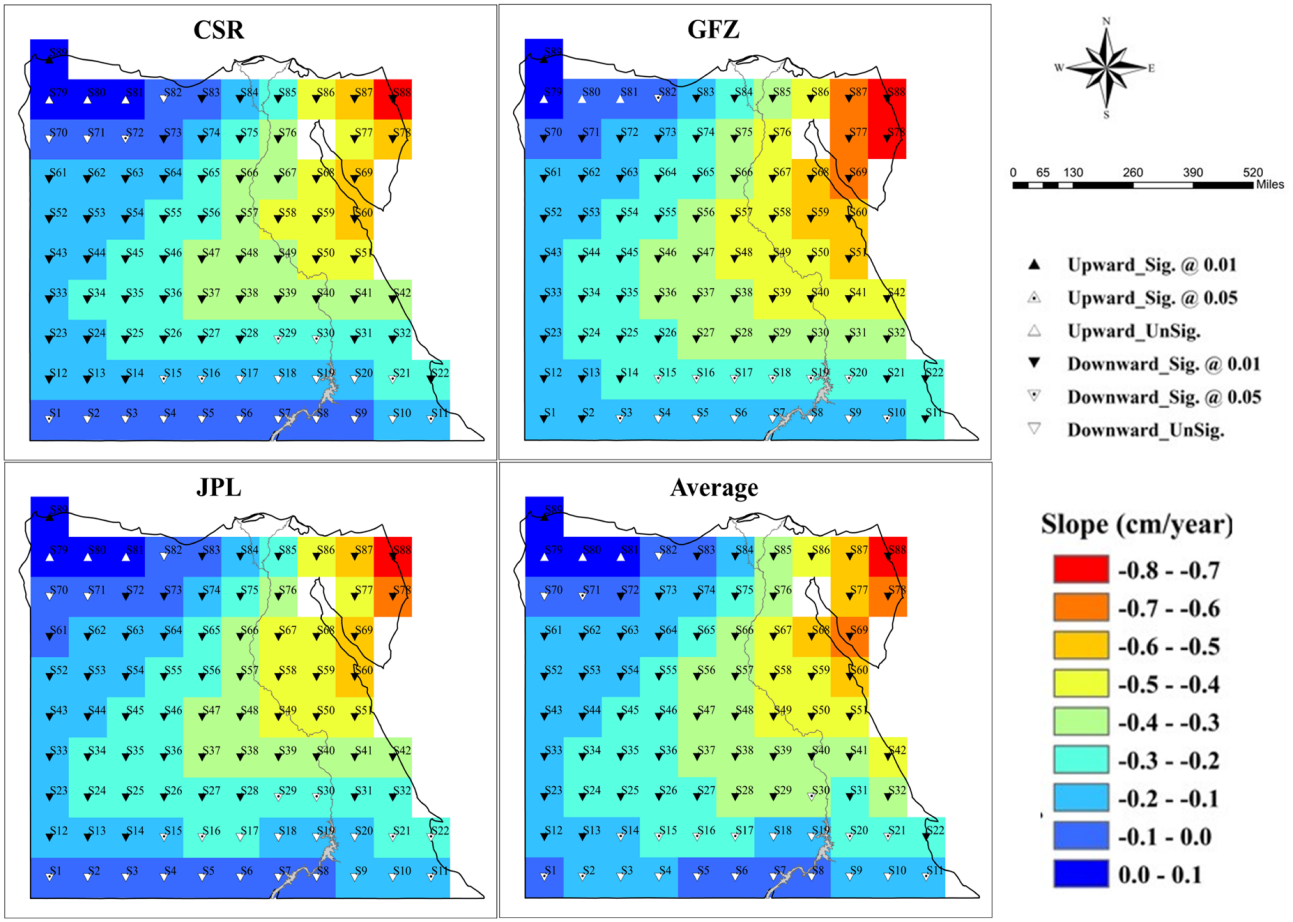
Spatial pattern of trends in terrestrial water storage from 2003 to 2021 throughout Egypt

The obtained time series of TWS variations were analyzed in 89 grids all over Egypt based on the solutions from three sources (i.e., CSR, JPL, and GFZ) in addition to their average. The annual averages of the extracted monthly time series from 2003 to 2021 were investigated and posted in a GIS environment. The ΔTWS values of the three solutions and their average have turned into a negative sign in almost all grids since the year of 2009. The negative sign denotes a loss in the TWS. Figure 2 depicts the spatial pattern of the analyzed trend values of TWS, estimated by linear regression analysis over the entire investigated period, where almost all time series showed a negative slope value. For the four data sets, a significant upward trend is observed in one grid (S89). Only four grids (S79, S80, S81, and S89), located at the north-western corner of Egypt, have shown positive slopes ranging from 0.01 to 0.05 cm/year according to the CSR and JPL solutions, as well as the average of the three solutions. While the solution from the GFZ showed a monotonic slope at S79 and S81, an upward significant trend of 0.04 cm/year at S89, and a slight upward slope of 0.02 cm/year at S80. In contrast, the maximum downward was detected at the eastern portions within the Sinai Peninsula, i.e., S88 showed significant negative trends estimated as −0.71 ± 0.03 cm/year, −0.72 ± 0.03 cm/year, −0.77 ± 0.03 cm/year, and −0.73 ± 0.03 cm/year, respectively, for the three solutions (CSR, JPL, and GFZ) and their averaging. All the grids within the middle portion of the country experience a downward trend ranging from 0.3 to 0.5 cm/year. While the grids along the western border showed a downward trend of about 0.2 cm/year. Grids along the south border experience a slight downward slope affected by the recharge from Lake Nasser (the High Dam’s reservoir). These storage variations refer to changes in groundwater, and soil moisture; however, the groundwater is believed to be the main component throughout the case study.

### Temporal variations in SM

**Fig. 3:**
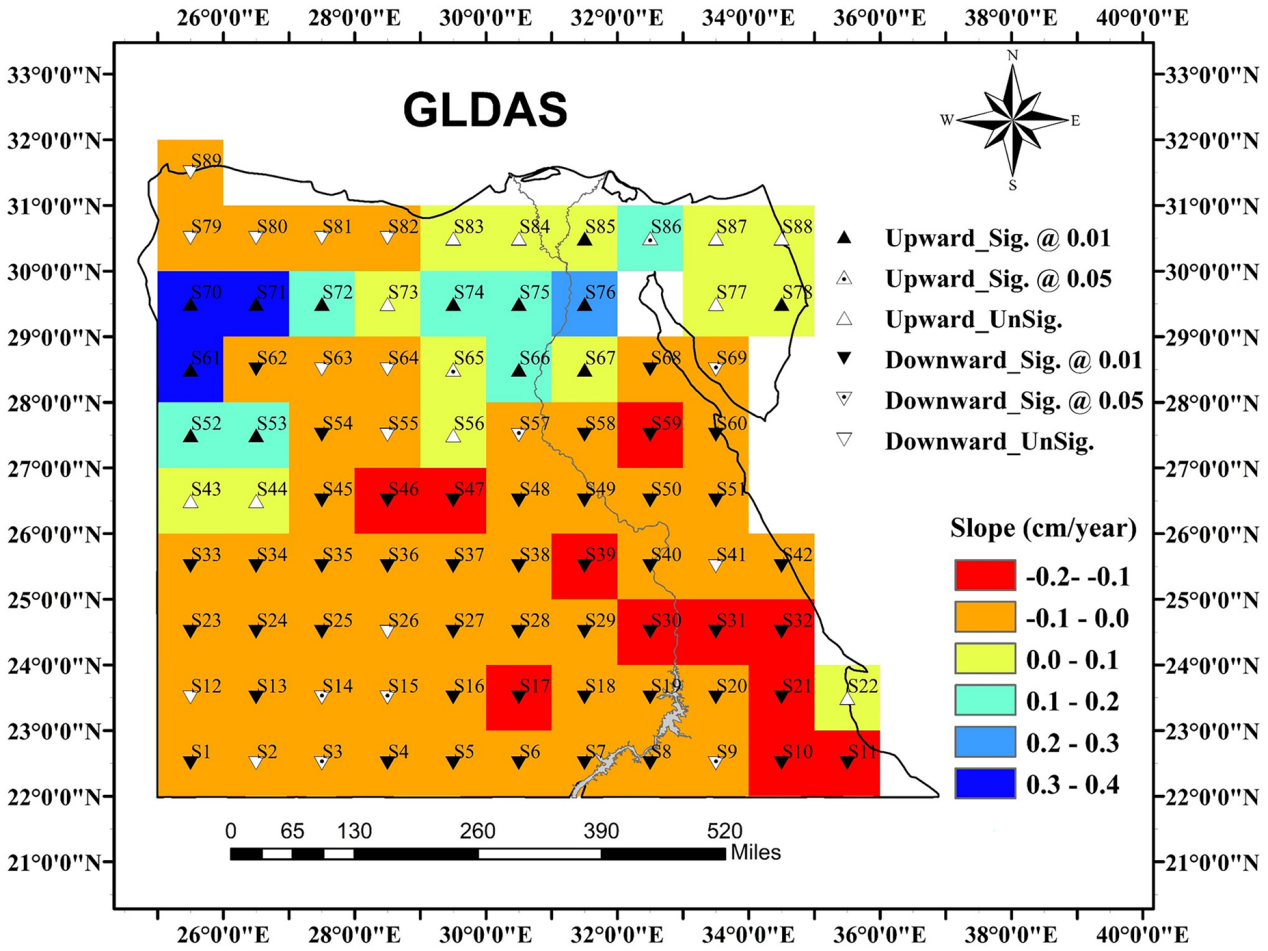
The trend of change in the soil moisture content for the highest two meters over Egypt

The GLDAS hydrological model integrates a large quantity of observed data including total column soil moisture (SM). The SM through the top 200 cm of the soil depth was extracted to represent the unsaturated TWS change in the study area. The SM storage is fairly (stable), and the amplitudes of the four layers are quite similar; however, a little dampening at the upper 50 cm, where shallow-rooted crops are penetrated, was recognized. The spatial trend pattern of GLDAS-derived SM is shown in Fig. 3. Changes in the SM are less than that observed in GRACE-inferred TWS. Almost (75%) of the grids showed a significant downward trend with a gentle slope that does not exceed 0.1 cm/year. Accordingly, it is proved that the TWS loss mainly occurs in the saturated zone due to groundwater pumping. A significant increase in the SM by about 0.1–0.3 cm/year was observed within the Nile delta and its fringes. This increase in soil water content within the unsaturated zone is attributed to flood irrigation agriculture. Similarly, the increase in SM at some grids in the Western Desert is attributed to the ongoing cultivation activities depending on natural flowing springs through the desert oasis (Elmansy et al., 2020). The upward trend in SM observed within the Sinai Peninsula may be attributed to the frequent flash flood events (Gado, 2020).

### Temporal variations in GWS

**Fig. 4:**
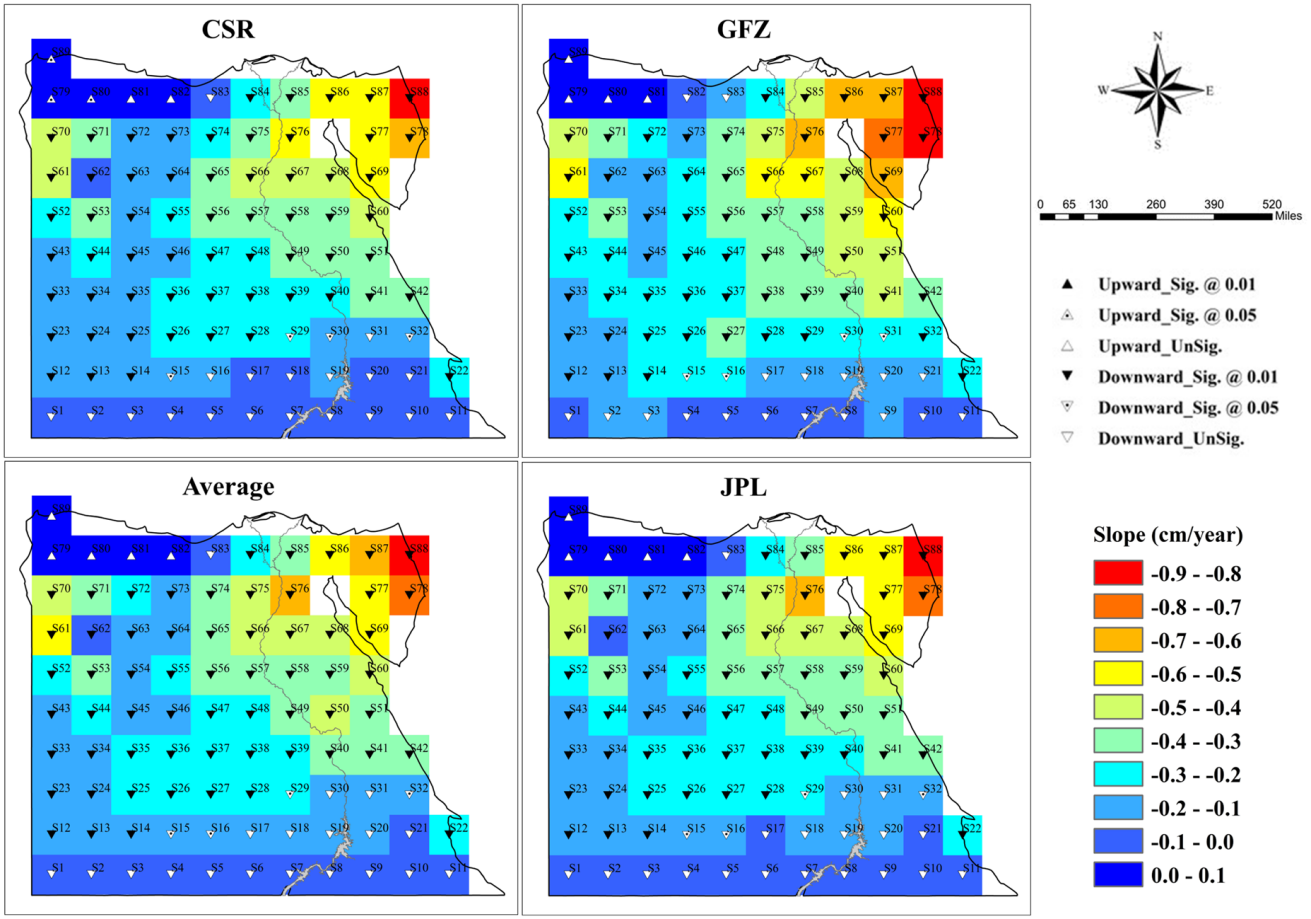
Spatial pattern of trends in groundwater storage from 2003 to 2021 throughout Egypt

Changes in GWS were inferred as the differences between changes in GRACE-derived TWS and GLDAS-simulated SM, believing that the other two water components (i.e., snow and surface water) are typically null, and their contributions to the long-term trend of TWS in Egypt are negligible. The spatial pattern of GWS trends for Egypt from 2003 to 2021 is depicted in Fig. 4. Similar to the results of the TWS time-series trend analysis, the GWS exhibited a decreasing trend across almost all grids but with different trend values. Moreover, the map of the spatial trend of the ΔGWS time series resembles that of the ΔTWS time series. Grids in the northwestern corner of Egypt, which represent the spatial extent of the Salloum plateau, showed positive trends. The reasons for the upward trends that prevailed over the Salloum plateau are: (1) there is no groundwater withdrawal within the Salloum plateau because the local population (the Bedouin community) depends mainly on rainfall which records an annual average of 63 mm (Gado, 2020), (2) rainfall infiltration into the soil, and (3) the seawater invasion from the Mediterranean Sea. In the contrast, the most obvious decline of groundwater storage is noticed in plots that occupied the northeastern part (Sinai desert) of the country. The main reason for this region’s considerable reduction in GWS is evapotranspiration and increasing groundwater extraction either in the Sinai or the adjacent Negeb desert (Negev) in Palestine.

**Fig. 5:**
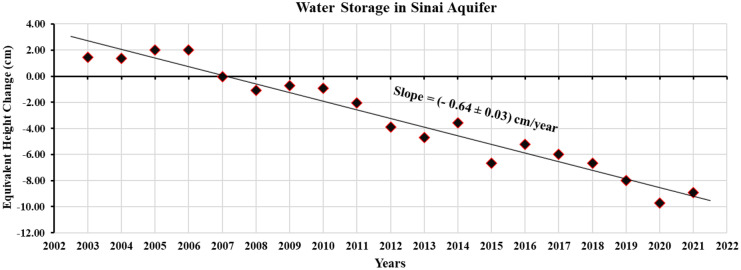
Annual changes in groundwater storage throughout the Sinai Peninsula, as estimated from the GRACE data

The area-weighted averaging technique was used to process the annual time series of GWS changes throughout the Sinai Peninsula showing a significant decrease in the storage by 388 Mm^3^/year, Fig. 5. This storage loss is mainly attributable to groundwater pumping. Indeed, the groundwater in the Sinai Peninsula exists as a thin water lens (i.e., perched aquifers) and, therefore, is highly susceptible to abstraction-triggered stresses (Gad et al., 2015). The pumped water is utilized to meet various needs as there is no other source of freshwater. This result is supported by the findings of many studies attributing water salinization and wells drying up to the over-pumping to depleted levels (Bekhit, 2015; Eissa et al., 2013; El-Bihery, 2009; Gad & Khalaf, 2015).

**Fig. 6:**
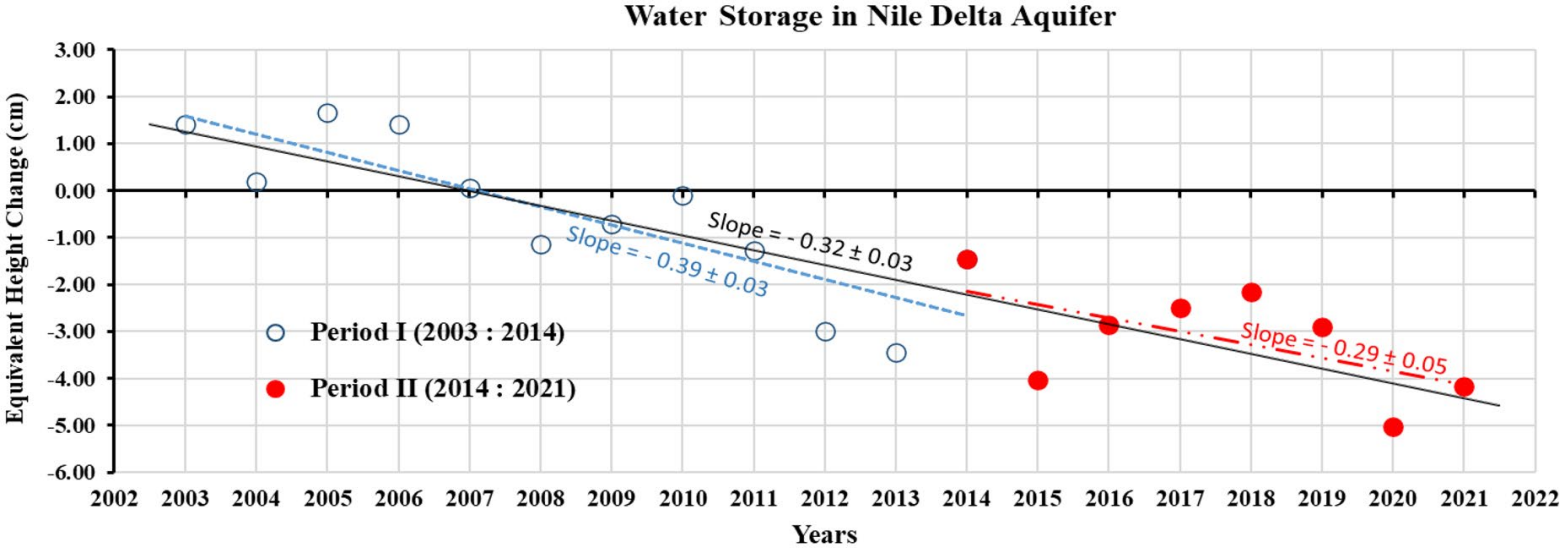
Annual changes in groundwater storage over the Nile Delta aquifer, as estimated from the GRACE data

Following the processed annual changes of the Nile Delta aquifer storage, a significant decreasing trend of (0.32 ± 0.03) cm/year is detected along the investigated period (2003–2021), Fig. 6. The average storage loss is estimated as 104.5 Mm^3^/year. These derived results are consistent with the literature (e.g., Mabrouk et al., 2018; Sefelnasr & Sherif, 2014; Sobeih et al., 2017) to confirm the Nile Delta Aquifer has experienced a significant storage loss in the last two decades. Yet, this depletion rate is lower than the average extraction rate, reported as about 3.16 Bm^3^/year (Armanuos & Negm, 2019). That is attributed to higher recharge rates from the Nile River, irrigation canals, and precipitation. Intruded seawater into the aquifer also contributes to compensating for a part of the groundwater withdrawal (Mabrouk et al., 2013). Recharges from the two Nile branches and seepage from irrigation networks are estimated at 2.6 km^3^/year (RIGW, 2003), in addition to an average of 0.42 km^3^ seawater annually intrudes into the aquifer (Armanuos et al., 2017). The average annual natural discharge toward the Moghra aquifer is 85 Mm^3^/year (Sayed et al., 2020). Figure 6 indicates that the storage loss was not significant until 2007, thereafter the ΔGWS has considerably changed. Subsequently, the abstraction rate has increased dramatically, exceeding the recharge rate, which caused a significant decrease in the aquifer storage. The time series of the Nile Delta aquifer’s storage showed a change point in the year 2014. The slope changed from −0.39 (2003–2014) to −0.29 (2014–2021). This is explained by the recharging increase to the aquifer due to expansion in agricultural activities and accelerated seawater intrusion owing to sea level rise.

**Fig. 7:**
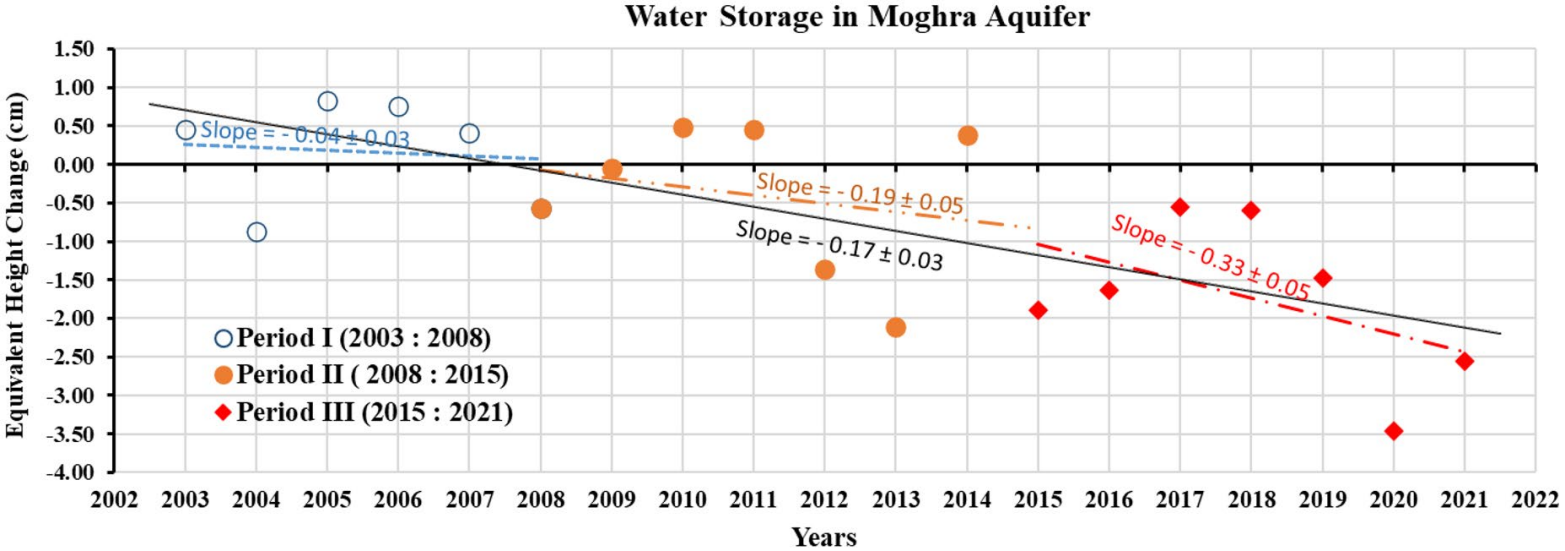
Annual changes in groundwater storage in the Moghra aquifer, as estimated from the GRACE data

Figure 7 illustrates the time series of annual changes in GWS of the Moghra aquifer that was derived as GRACE-TWS series minus GLDAS-SM series. The figure showed a persistent decrease in the aquifer storage since 2015 when the aquifer is equipped for irrigation of newly cultivated lands. For the period from 2003 to 2008, a slight decreasing trend of (0.04 ± 0.03) cm/year has been detected. During this period, the aquifer was roughly considered an untapped aquifer (Morad et al., 2014). The decrease in aquifer storage during the first investigated period is mainly attributed to evapotranspiration (Sayed et al., 2020). During the second period (2008–2015), the aquifer storage decreased at a rate of (0.17 ± 0.03) cm/year. The increase in storage loss is due to water withdrawal through some private shallow wells that partially penetrate the aquifer (Abdel Mogith et al., 2013). The loss in the aquifer storage during the first two periods is 32 and 135 Mm^3^/year, Fig. 7. The loss doubled within the third period (2015–2021), reaching 262 Mm^3^/year. This huge amount of pumped water is exploited to irrigate a wide reclaimed area in the Western Desert within the national reclamation project.

**Fig. 8:**
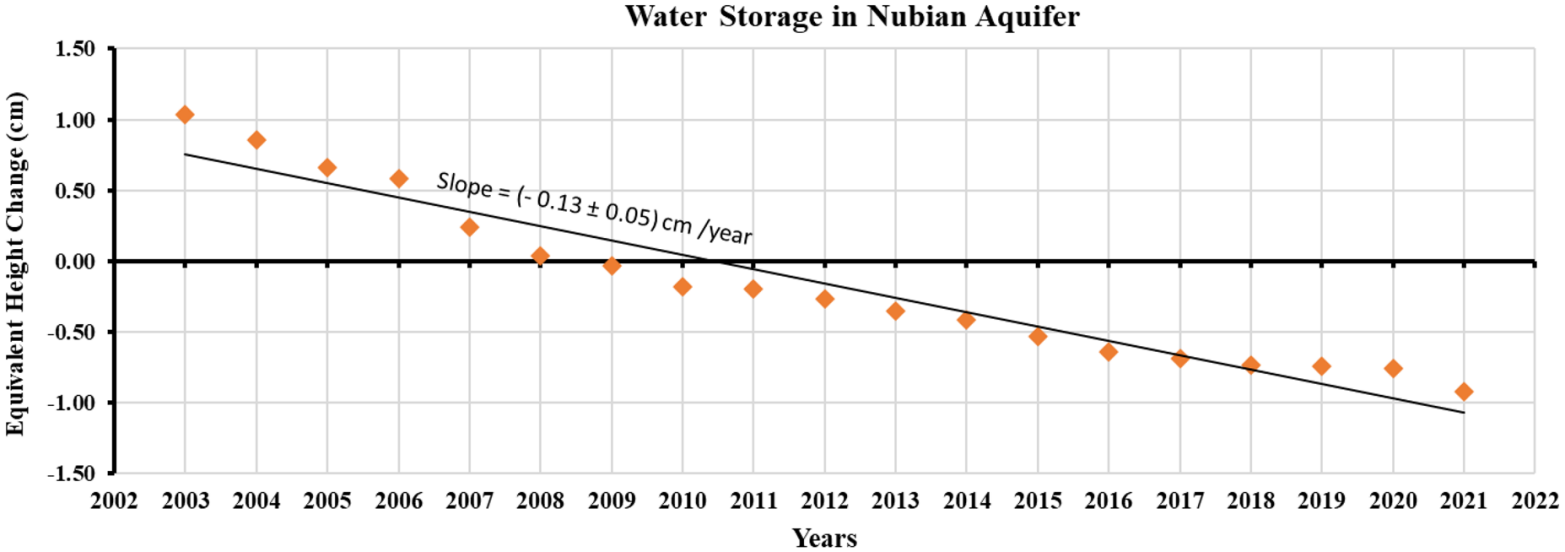
Changes in groundwater storage in the Nubian aquifer, as estimated from GRACE data

The extensive rural development depending on the Nubian aquifer’s potentiality in the Western Desert significantly dropped its storage by about 2.5 cm in the last two decades, Fig. 8. Considering the large areal extensions of the Nubian aquifer, the extracted groundwater quantity is about 7.25 km^3^. It may be argued these estimates underestimate the observed huge drawdown in the groundwater table. Nevertheless, it is worth mentioning the fact that the cone of depression in piezometric heads is limited within the wells fields (Elmansy et al., 2020). Moreover, the aquifer has initiated the recovery of its reserve from Lake Nasser. As the aquifer system is hydraulically connected with Lake Nasser which recharges it by a huge water quantity (Aly et al., 2019). However, the recharge is controlled by the difference between the lake water level and the aquifer water table. The findings confirm that there is substantial room for rural development depending on the huge storage of the Nubian aquifer.

## Findings verification against in situ observations

A spatial match between the grids that have indicated a storage decreasing trend according to GRACE-based GWS and plots of high groundwater pumping rate. That confirms the GRACE’s ability to monitor groundwater depletion in Egypt. The results showed that the Nile Delta aquifer is subjected to significant storage loss since 2007. These findings can be evidenced by hydrogeological observations showing a noticeable drawdown in water levels throughout the aquifer. However, the GRACE-derived storage losses of (104.5 ± 9.8) Mm^3^/year is not coincide with the apparent high extraction rates from the aquifer. The difference is resulting from replenishment sources and intruded seawater that partially compensates for the groundwater extraction. Armanuos and Negm, (2019) stated that the net budget of the system equals − 0.221 Km^3^/year. This value is consistent with the storage loss estimated as GRACE-TWS minus GLDAS-SM. However, considering the slight changes in surface water (i.e., irrigation canals) throughout the Nile Delta may improve the accuracy of storage decline estimation.

The results showed a moderate reduction of the GWS occurred in the fossil aquifers in the Western Desert where groundwater is overused. The GRACE-derived estimation was able to represent the actual three stages of groundwater exploitation in the Moghra aquifer (Gomaa et al., 2021). The aquifer was roughly untapped until 2008. After that, the aquifer experienced a rationed pumping rate causing a storage loss of about 1.5 Km^3^ during the period from 2009 to 2015. The development projects inaugurated in 2016 have multiplied pumping from the Moghra aquifer by nearly eight times; consequently, the aquifer has lost about 2.3 Km^3^ of its reserves from 2017 to 2021. Such intensive withdrawal threatens the sustainability of the ongoing agribusiness activities depending on the aquifer as the sole water supply. Furthermore, a significant groundwater depletion of 0.4 Km^3^/year was detected throughout the Sinai Peninsula. The resulting drawdown has triggered the upwelling of deep saline groundwater in wells near the coast (Bekhit, 2015; Eissa et al., 2013; Omran, 2019). This study urges the close monitoring of any future groundwater pumping in Sinai to avoid (or limit) such effects. In the contrast, the huge storage of the Nubian aquifer encourages future expansion in cultivation projects. The regional representation of the Nubian aquifer system indicated the head drawdown is concentrated around the wells field and the storage experienced a slight loss and may be recoverable.

## Conclusions

Egypt is located in one of the most water-stressed areas globally. It has relied increasingly on groundwater reserves to compensate for the deficit in freshwater resources and to supply the bulk of water demands for rural development projects. This work combined data from GLDAS and GRACE to track alteration in the terrestrial water components throughout Egypt, where scarce observations limit hydro-climatological studies. Recently released GRACE time-variable gravity accurate solutions were utilized to analyze TWS variability in Egypt. The TWS experienced a significant loss rate throughout almost Egypt, with slight differences between the three GRACE-data processing sources. Change in the SM was less than that in the saturated zone, affirming that depletion occurs mainly in the saturated storage due to groundwater pumping. Changes in GWS were then calculated as the difference between GRACE-derived TWS and GLDAS-simulated SM. GRACE signals indicate a significant reduction of the GWS almost over all of Egypt. The decreasing trend of GWS is even more remarkable in some locations, which is attributed to groundwater over-pumping and evapotranspiration from shallow aquifers. On the contrary, the upward trend along the Mediterranean Sea is attributed to saltwater invasion into the north coastal aquifer.

The research’s findings highlight the main role of observing aquifers’ storage in management plans and decision-making processes to achieve resource sustainable management. In this context, the utilization of information acquire by GRACE satellites as an additional regularization mechanism to constrain a regional groundwater model over a large-scale aquifer would improve the simulation results (Hu & Jiao, 2015; Sun et al., 2012). However, applying a de-striping or/and smoothing filter may be a prerequisite for a successful combining of GRACE water storage anomalies data into such models (Ahmed et al., 2014). The filtering approaches contribute to removing the correlated errors (stripes) and reducing the risk of poor consistency and comparability of data from different sources. Moreover, using GRACE mascon solutions of a high spatial resolution may be robust. GRACE data validation against in situ well observations, if available, is urged to assess uncertainty and to apply a reliable bias correction technique to improve the skill of the GRACE-based approach.

## Data Availability

The data that support the findings of this study are available from the corresponding author, upon reasonable request.
